# Use of CatBoost algorithm to identify the need for surgery in infants with necrotizing enterocolitis

**DOI:** 10.3389/fped.2025.1465278

**Published:** 2025-02-21

**Authors:** Xinyun Jin, Wenqiang Sun, Yihui Li, Yinglin Su, Lingqi Xu, Xueping Zhu

**Affiliations:** ^1^ Department of Neonatology, Children’s Hospital of Soochow University, Suzhou, China; ^2^ Clinical Pediatrics School, Soochow University, Suzhou, China; ^3^ Department of Neonatology, Wuxi Children’s Hospital, Wuxi, China

**Keywords:** necrotizing enterocolitis, surgical NEC, risk factors, CatBoost machine learning model, GUI interface

## Abstract

**Background:**

Early identification of infants with necrotizing enterocolitis (NEC) at risk of surgery is essential for an effective treatment. This study aims to clarify the risk factors of surgical NEC and establish a prediction model by machine learning algorithm.

**Methods:**

Infants with NEC were split into two groups based on whether they had surgery or not. Clinical data was collected and compared between the groups. Variables were analyzed with one-way logistic regression and predictive models were built using logistic regression and CatBoost algorithm. The models were evaluated and compared using Receiver Operating Characteristic (ROC) curves and feature importance. Feature importance was ranked using the SHapley Additive exPlanation method and model optimization was performed using feature culling. Final model was selected and a user-friendly GUI software was created for clinical use.

**Results:**

The Catboost model performed better than the logistic regression model in terms of discriminative power. An interpretable final model with 14 features was built after the features were reduced according to the feature importance level. The final model accurately identified Surgicel NEC in the internal validation (AUC = 0.905) and was translated into a convenient tool to facilitate its use in clinical settings.

**Conclusions:**

Catboost machine learning model related to infants with surgical NEC was successfully developed. A GUI interface was developed to assist clinicians in accurately identifying children who would benefit from surgery.

## 1 Introduction

Necrotizing enterocolitis (NEC) is an acute critical bowel disease commonly occurring in preterm infants. The incidence of NEC in preterm and very low birth weight neonates ranges from 2% to 13%, with an average mortality rate of 20%–30% (1, 2). The survival rate of preterm infants has significantly increased with the improvement of perinatal medicine and neonatal critical care. The incidence of NEC has also increased, and has become one of the diseases that seriously threaten the life and health of preterm newborn. However, there is a lack of specific treatment for this disease. The conservative medical treatment is the mainstay, and some severe cases require surgical treatment, but the mortality rate of children treated with surgery is as high as 50% (3).

Notably, infants requiring surgical intervention often present with more advanced and severe disease, which significantly contributes to their elevated mortality rates, poorer long-term prognosis, and diminished quality of life compared to those managed with conservative medical care (4, 5). This represents a substantial clinical and emotional burden for both the patients and their families (6). The etiological hypothesis of NEC is still incomplete, and its pathogenesis is highly complex and multifactorial. It is currently associated with an excessive inflammatory response and necrosis of the intestinal tissue due to multiple perinatal risk factors that act on the immature intestinal tract of preterm infants, including perinatal asphyxia, antibiotic use, no breastfeeding, infections, and abnormal fixation of the intestinal flora (3, 7). Therefore, the early identification of this disease and the need for surgery are essential, as they enable timely intervention in patients with severe disease, reduce surgical risk in patients with NEC, and improve the overall prognosis.

Currently, most of the studies focus on risk prediction models for the occurrence and severity of NEC, and only few studies are available on the models for the early prediction of the need of surgery in NEC patients. Moreover, the existing prediction models are heterogeneous, the indicators included are not comprehensive enough, and the clinical operability and accuracy are not good. The rapid development of precision medicine has allowed the use of machine learning methods featuring deep analysis in the construction of clinical prediction models, but few reports are available on the construction of models to predict whether NEC patients need surgery. Furthermore, the existing models show limitations, mainly represented by low predictive efficacy, poor operability, and insufficiently comprehensive incorporation of metrics (8–10).

Therefore, in this work, more comprehensive perinatal clinical data, laboratory tests and imaging examinations were included according to the pathophysiological mechanism of NEC, which were analyzed using machine algorithms. A scientific risk prediction model was then constructed by selecting the risk variables with the greatest predictive efficacy according to the importance of the characteristics and clinical operability. Thus, a theoretical foundation was provided to further improve the clinical identification of high-risk NEC, ensure timely treatment, and prevent further deterioration of the disease for further improving the long-term quality of life of children.

## 2 Methods

### 2.1 Study population

Clinical data of all children with NEC admitted to the neonatal unit of Children's Hospital of Soochow University from 1 January 2012 to 31 December 2021 were retrospectively analyzed, and the study was pre-approved by the institutional review board. The inclusion criteria for this study were the modified Bell staging ≥ stage II. The exclusion criteria were the following: children with immunodeficiencies, inherited metabolic disorders, congenital malformations of the intestine, and patients with pneumoperitoneum detected during preoperative examination, regardless of the presence of NEC. Additionally, cases of infants transferred from other hospitals who underwent a change in treatment from conservative to surgical without adequate clinical data were excluded. Enrollment in all cases was independently reviewed by two experts, with a third expert consulted in case of disagreements. All clinical and demographic data utilized in this study were retrospectively extracted from medical records. These data encompass patient characteristics and clinical parameters recorded at the time of NEC diagnosis, as well as subsequent clinical deterioration or preoperative status prior to surgical intervention. The study was approved by the Ethics Committee of the Children's Hospital of Soochow University (2023CS189; Suzhou, China), informed consent was waived by the ethics committee. The study was adhered to the tenets of the Declaration of Helsinki.

### 2.2 Data collection and processing

Variables including general data, maternal prenatal general data, underlying disease and comorbidities, treatment before diagnosis of NEC, laboratory testing and imaging, nutritional management before NEC diagnosis were collected and analyzed. Cases with more than 50% missing data for any variable were excluded from the analysis. Additionally, variables with more than 50% missing values were also excluded. For the remaining missing values, mean imputation was applied for continuous variables, and mode imputation was used for categorical variables.

Certain indicators, such as WBC, PLT, lymphocyte count, CRP, and procalcitonin, were categorized due to their clear normal ranges in neonates on the first day of life, facilitating the detection of abnormalities. In contrast, variables like hemoglobin and albumin were kept as continuous data to better capture subtle changes and their correlation with disease progression. This approach also allows the CatBoost algorithm to model nonlinear relationships more effectively, thereby improving the model's predictive accuracy.

### 2.3 Outcome

Binary labels were constructed based on the dataset after dividing the population into two groups: conservative treatment NEC and surgical NEC using the revised Bell staging to define the different treatment modalities in the model. The conservative group involved conservative management, which included cessation of feeding, parenteral nutrition, and empirical antibiotic therapy. Surgical indications were defined by radiographic evidence of significant bowel perforation (pneumoperitoneum) or, in the absence of imaging findings, clinical deterioration despite prolonged conservative management. Infants who died in the conservative group were classified into the surgical group to mitigate potential bias, as these infants were likely candidates for surgery but were unable to receive it in a timely manner due to the progression of their condition. Surgical intervention was defined as including abdominal drainage and exploratory laparotomy. If the disease outcome was in the surgical group, the label was 1, otherwise, the label was 0. This binary labelling approach was used for both the training set and the validation set.

### 2.4 Model development and comparison

The goal of this study was to accurately predict whether a child would progress to NEC requiring surgery based on the clinical data (clinical indicators, test and examination results). The outcome was a binary categorical problem in which the target variable was the binary short-term progression of NEC. The explanatory variables were the parameters included in the measurement. Prediction was performed using two machine learning algorithms, including LR and CatBoost. The LR and Catboost models were implemented using Python packages.

The dataset was randomly divided into two subsets: 70% of the data was allocated for training the models, while the remaining 30% was reserved for the validation cohort. This division allowed for internal validation and ensured that model evaluation was conducted on data not seen during training, thus minimizing the risk of overfitting.

Model performance was assessed exclusively on the validation cohort to evaluate the models' generalizability to unseen data. Key performance metrics included the area under the ROC curve and its corresponding 95% confidence intervals. The AUC score was used to quantify the models' ability to distinguish between infants requiring surgical vs. conservative treatment for NEC. Additionally, other performance measures, such as sensitivity, specificity, and accuracy, were calculated to provide a comprehensive evaluation of the models' predictive capabilities.

### 2.5 Explanation of the predictions

A modern tool for interpretable AI called SHapley Additive exPlanations (SHAP) was used to determine the most important factors and their contribution to the prediction. This so-called SHAP value enabled the interpretation of the results of a machine learning model using game theoretic concepts: the Shapley value. The SHAP value quantified the marginal contribution of each feature to the final prediction. This study used the CatBoost machine learning algorithm for the final model, which was interpreted using the SHAP value.

### 2.6 Development of web tools

The Graphics User Interface (GUI) was developed using the Python-based Tkinter module.

### 2.7 Statistical analysis

Statistical analysis was performed using Python version 3.6.5 and SPSS 26.0 software. Count data were expressed as cases and percentages (%), and the comparison between the two groups was made using the *χ*^2^ test or Fisher's exact probability method. Measurement information was expressed as median (interquartile spacing) [M (P25, P75)], and comparison between groups was performed using the Mann–Whitney *U* test. Covariance was assessed on the results of univariate logistic regression analysis (all tolerances ≥ 0.1 and VIF values ≤ 10). Variables with statistically significant difference in univariate logistic regression analysis were included in logistic regression analysis and Catboost regression analysis. Predictive efficacy was evaluated using the AUC, and the optimal threshold was established by maximizing the Youden index (sensitivity + specificity − 1). A two-tailed value of *P* < 0.05 was considered statistically significant.

## 3 Result

### 3.1 Demographics

**Figure 1:**
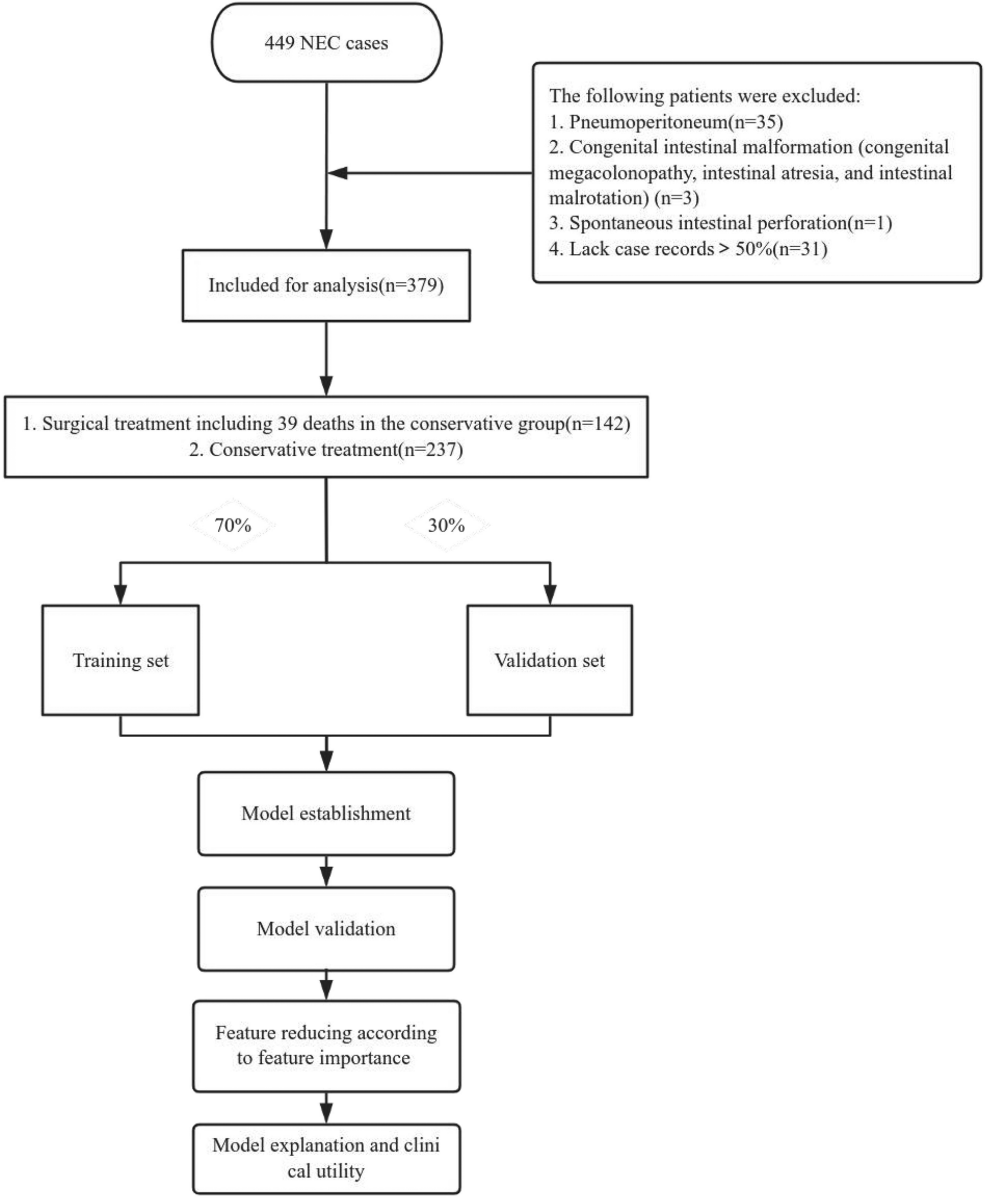
Flow chart of the study design. NEC, Necrotizing enterocolitis.

This study involved 449 NEC cases in the cohort, all Bell stage II or higher, for predictive modelling. Thirty-five children with pneumoperitoneum on preoperative examination, thirty-one children with more than 50% missing case records, three children with congenital intestinal malformations (congenital megacolonopathy, intestinal atresia, and intestinal malrotation), and one child with spontaneous intestinal perforation were excluded during the study period. A total of 379 children were finally enrolled in this study, with 142 receiving surgical treatment (including 39 infants who died in the conservative treatment group) and 237 receiving conservative treatment. Details of the study design are shown in Figure 1. These children were randomly assigned to either the training or validation set (7:3). The clinical characteristics of the two groups of patients are listed in Table 1.

**Table 1:** Comparison of clinical characteristics between the two groups of patients.

Factors	Conservative NEC group (*n* = 237)	Surgical NEC group (*n* = 142)	Effect value	*P* value
General data				
Male [person (%)]	141 (59.49)	75 (52.82)	1.615	0.204
Birth weight [g, (x̄ ± s)]	2,151.43 ± 808.99	1,755.67 ± 821.41	4.583	**0**.**000**
Gestational age [w, (x̄ ± s)]	34.51 ± 3.53	32.11 ± 3.69	2.688	**0**.**000**
Premature infants [person (%)]	173 (73.00)	125 (88.03)	11.941	**0**.**001**
IVF [person (%)]	18 (7.59)	7 (4.93)	1.024	0.312
Small for gestational age [person (%)]	64 (23.44)	30 (21.13)	1.645	0.200
Apgar score at 1 min < 7 [person (%)]	31 (13.08)	27 (19.01)	2.412	0.120
Apgar score at 5 min < 7 [person (%)]	17 (7.17)	14 (9.86)	0.853	0.356
Utero distress [person (%)]	19 (8.02)	10 (7.04)	0.119	0.730
Maternal prenatal general data				
Parental age [Year, M (P25, P75)]	29 (26, 32)	29 (26, 33)	−1.056	0.292
Cesarean delivery [person (%)]	106 (44.73)	80 (56.34)	4.791	**0**.**029**
Multiparous pregnancies [person (%)]	49 (20.68)	35 (24.65)	0.812	0.367
Antenatal corticosteroids treatment [person (%)]	127 (53.59)	95 (66.90)	15.01	**0**.**000**
Premature rupture of membranes ≥18 h [person (%)]	31 (13.08)	33 (23.24)	6.530	**0**.**011**
Gestational hypertension [person (%)]	42 (17.72)	24 (16.90)	0.042	0.839
Gestational diabetes mellitus [person (%)]	33 (13.92)	18 (12.68)	0.037	0.847
Hypothyroidism [person (%)]	13 (5.49)	6 (4.26)	0.296	0.586
Intrahepatic cholestasis [person (%)]	10 (4.22)	3 (2.11)	0.693	0.424
Umbilical cord bypass [person (%)]	16 (5.65)	2 (1.85)	2.984	0.084
Antenatal febrile [person (%)]	11 (4.64)	4 (2.82)	3.245	0.072
Maternal anemia [person (%)]	7 (2.95)	4 (2.82)	0.000	1.000
Hydramnios [person (%)]	2 (0.84)	2 (1.41)	0.000	0.999
Oligohydramnios [person (%)]	17 (7.17)	8 (5.63)	0.341	0.559
Turbid amniotic fluid of degree III [person (%)]	6 (2.53)	2 (1.41)	0.135	0.713
Placental abruption [person (%)]	21 (8.86)	12 (8.45)	0.091	0.891
Underlying disease and comorbidities				
Frequent sleep apnea [person (%)]	18 (13.14)	42 (29.58)	32.202	**0**.**000**
Type-2 respiratory failure [person (%)]	29 (12.24)	48 (33.80)	25.512	**0**.**000**
Hypokalemia [person (%)]	20 (8.44)	19 (13.38)	2.349	0.125
Hyponatremia [person (%)]	11 (4.64)	19 (13.38)	9.303	**0**.**002**
Anemia [person (%)]	40 (16.88)	46 (32.39)	12.187	**0**.**000**
hsPDA [person (%)]	51 (21.52)	16 (11.27)	2.614	0.106
Use of drugs to close PDA [person (%)]	2 (1.41)	14 (9.86)	15.689	**0**.**000**
NRDS [person (%)]	30 (12.66)	45 (31.69)	20.263	**0**.**000**
ASD [person (%)]	135 (56.96)	76 (53.52)	0.426	0.514
Sepsis [person (%)]	22 (9.28)	41 (28.87)	26.483	**0**.**000**
Treatment before diagnosis of necrotizing enterocolitis				
Alveolar surfactant [person (%)]	34 (14.35)	41 (28.87)	11.806	**0**.**001**
Blood transfusion ≥3 times [person (%)]	7 (3.04)	6 (4.23)	0.135	0.714
Prophylactic antibiotics [person (%)]	13 (5.49)	4 (2.82)	1.467	0.214
Aminophylline [person (%)]	3 (1.27)	12 (8.45)	12.059	**0**.**001**
Caffeine [person (%)]	8 (3.38)	18 (12.68)	12.059	**0**.**001**
Plasma transfusion [person (%)]	30 (12.66)	16 (11.27)	0.161	0.688
Vasoactive drugs [person (%)]	8 (3.38)	27 (19.01)	25.909	**0**.**000**
Laboratory testing and imaging				
White blood cell (WBC) counts (1d) [person (%)]				
>25 × 10^9^/L	2 (0.84)	1 (0.70)	0.000	1.000
<5 × 10^9^/L	39 (16.46)	62 (43.66)	33.624	**0**.**000**
Hemoglobin(1d) [g/L, (x̄ ± s)]	136.67 ± 31.90	128.79 ± 30.98	2.352	**0**.**019**
PLT < 100 × 10^9^/L (1d) [person (%)]	25 (8.83)	20 (18.52)	9.935	**0**.**002**
Neutropenia [person (%)]	25 (10.55)	11 (7.75)	0.811	0.368
Lymphocyte count <2.0 × 10^9^/L (1d) [person (%)]	97 (34.28)	72 (66.67)	38.948	**0**.**000**
Albumin (1d) [g/L, (x̄ ± s)]	32.60 ± 3.45	29.90 ± 4.34	6.313	**0**.**000**
Eosinophil proportions > 5% (1d) [person (%)]	32 (13.50)	16 (11.27)	0.401	0.527
CRP ≥ 20 mg/L(1d) [person (%)]	26 (10.97)	27 (19.01)	4.776	**0**.**029**
PH < 7.25 (1d) [person (%)]	2 (0.84)	29 (20.42)	45.321	**0**.**000**
Base excess (1d) [mmol/L, M (P25, P75)]	−3.71 (−4.00, −2.10)	−3.71 (−6.85, −2.88)	−4.021	**0**.**000**
NLR values (1d) [M (P25, P75)]	1.53 (0.90, 2.52)	1.84 (0.89, 2.92)	−1.211	0.226
Intestinal rigidity	111 (46.84)	77 (54.23)	1.940	0.164
Intestinal wall thickening [person (%)]	92 (38.82)	75 (52.82)	7.060	**0**.**008**
Pneumatosis intestinalis [person (%)]	236 (99.58)	142 (100)	0.000	1.000
Portal vein gas [person (%)]	13 (6.71)	22 (15.74)	10.610	**0**.**001**
Nutritional management before NEC diagnosis				
Enteral feed initiation [d, M (P25, P75)]	1 (1, 2)	2 (1, 2.25)	−0.783	0.434
Breastfeeding initiation [person (%)]	27 (11.39)	6 (4.23)	5.738	**0**.**017**
Feeding speed >25 ml/(kg·day) [person (%)]	101 (42.62)	37 (26.06)	10.517	**0**.**001**
Feeding intolerance [person (%)]	52 (21.94)	44 (30.99)	3.841	0.050
Nutrient intake before NEC				
Amino acid per day [g/(kg·day), (x̄ ± s)]	1.62 ± 0.46	1.63 ± 0.41	0.334	0.739
Fat emulsions per day [g/(kg·day), (x̄ ± s)]	1.06 ± 0.55	1.10 ± 0.48	−0.563	0.574
Glucose per day [g/(kg·day), (x̄ ± s)]	6.70 ± 2.11	6.81 ± 1.62	−0.537	0.592
Average calories per day [kcal/(kg·day), (x̄ ± s)]	83.88 ± 16.93	82.73 ± 17.90	−0.565	0.572
Average total fluid volume [ml/(kg·day), (x̄ ± s)]	140.21 ± 17.89	139.51 ± 18.88	0.362	0.718
Weight growth rate [g/(kg·day), (x̄ ± s)]	6.96 ± 9.31	7.82 ± 4.60	−1.198	0.232

Comparison of demographic and clinical characteristics between conservative NEC and surgical NEC. IVF, *in vitro* fertilization; hsPDA, hemodynamically significant patent ductus arteriosus; PDA, patent ductus arteriosus; NRDS, neonatal respiratory distress syndrome; ASD, atrial septal defect; NLR values, neutrophil to lymphocyte ratio values.

Bold values denote the *p* values <0.05.

Among the general data, the gestational age and birth weight of NEC infants treated with surgery were significantly lower than those of NEC infants treated with conservative treatment. Specifically, the gestational age in the conservative treatment group ranged from 25.14 to 41.14 weeks (median: 31.57 weeks), while in the surgery group, it ranged from 27.00 to 41.71 weeks (median: 30.86 weeks). Regarding birth weight, the conservative treatment group showed a range from 750 g to 5,000 g (median: 2,000 g), while the surgery group ranged from 600 g to 4,500 g (median: 1,500 g). The proportion of preterm infants in the surgical group was higher than that in the conservative group. As regards the maternal prenatal general data, the proportion of cesarean delivery, antenatal corticosteroids treatment, and premature rupture of membranes ≥18 h in the surgery group were higher than those in the conservative group. As regards the underlying diseases or comorbidities before the diagnosis of NEC and treatment measures, the surgical treatment group had significantly higher proportion including frequent sleep apnea, type II respiratory failure, hyponatremia, anemia, use of drugs to close PDA, NRDS, sepsis, pulmonary alveolar surfactant, aminophylline, caffeine, and vasoactive drugs compared with the conservative group. As regards the laboratory results of blood test, the surgical treatment group had significantly higher parameters including WBC < 5 × 10^9^/L, PLT < 100 × 10^9^/L, lymphocyte count < 2.0 × 10^9^/L, CRP ≥ 20 mg/L, and PH < 7.25 compared with the conservative group. And the surgical treatment group had significantly lower parameters including hemoglobin, albumin, and base excess compared with the conservative group. The results of abdominal x-ray examination showed that patients with intestinal wall thickening and portal vein gas in the surgery group were more than those in the conservative group. In terms of nutritional management before NEC diagnosis, The surgical group had significantly fewer cases of initiating breast feeding before the onset of NEC and fewer cases of feeding speed > 25 ml/(kg·day) compared to the medical treatment group.

### 3.2 Risk factors

A significant difference in the following variables between the two groups was found by univariate analysis: birth weight, gestational age, premature infants, cesarean delivery, antenatal corticosteroids treatment, premature rupture of membranes >18 h, frequent sleep apnea, type II respiratory failure, hyponatremia, anemia, use of drugs to close PDA, NRDS, sepsis, alveolar surfactant, aminophylline, caffeine, vasoactive drugs, WBC < 5 × 10^9^/L, hemoglobin, PLT < 100 × 10^9^/L, lymphocyte count < 2 × 10^9^/L, albumin, CRP ≥ 20, PH < 7.25, base excess, intestinal wall thickening, portal vein gas, breastfeeding initiation, and feeding speed > 25 ml/(kg·day). The Collinearity test was performed on the results of univariate analysis, and the results showed that VIF values were less than 10, suggesting no significant collinearity between the variables.

### 3.3 Model development and performance comparison

**Figure 2:**
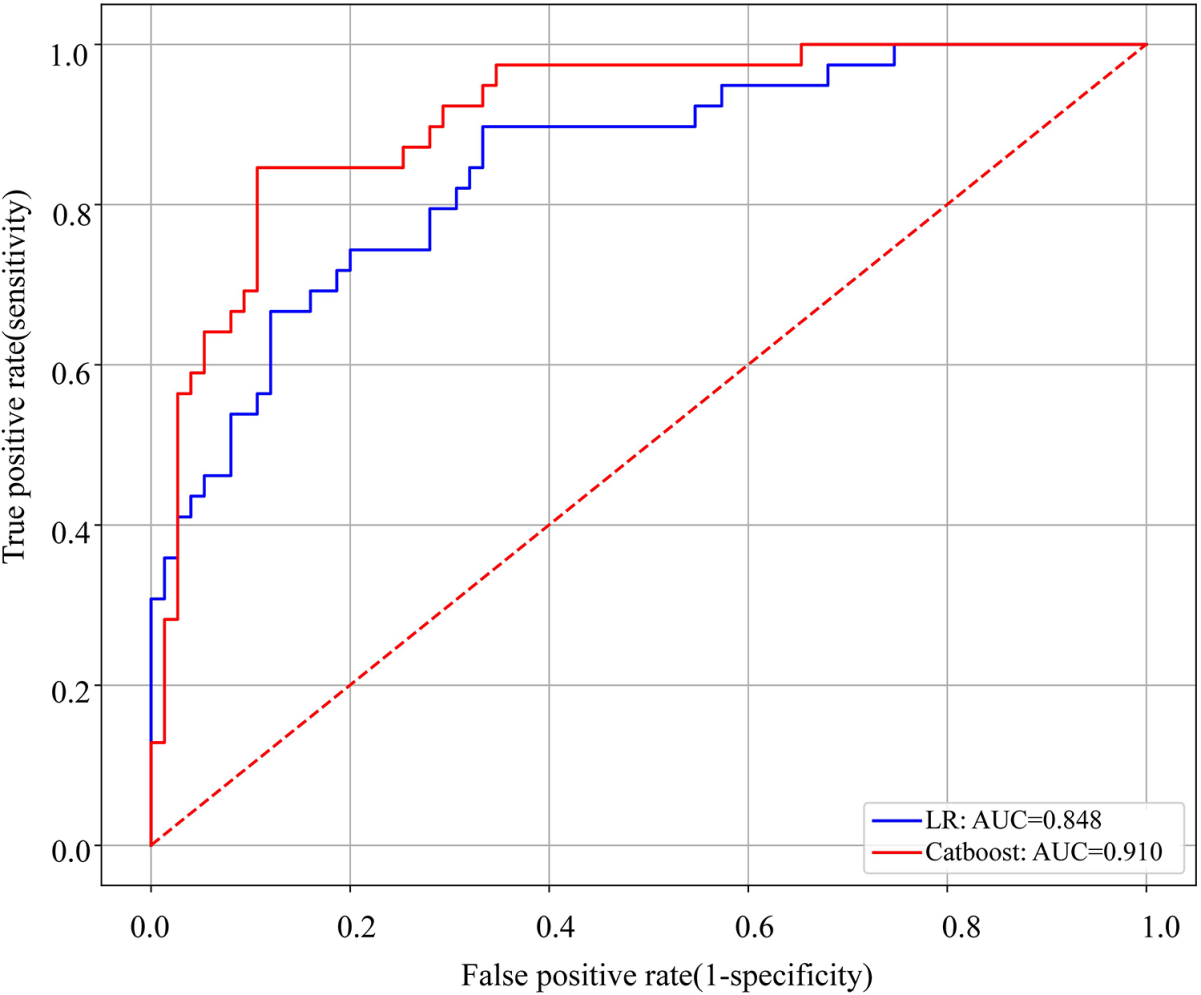
ROC curves of the Catboost and LR models to predict surgical NEC. AUC, area under the curve.

The Catboost model (AUC = 0.910) predicted NEC surgery better than Logistic regression (AUC = 0.848). The discriminative performance of these 2 models is shown in Figure 2, where the best cut-off value with the largest Youden index was taken to calculate the sensitivity, specificity, precision and accuracy. The sensitivity of Catboost and LR were 61.5% and 64.1%, the specificity was 82.6% and 82.5%, the precision was 0.857 and 0.735, and the accuracy was 0.833 and 0.798, respectively. The above results demonstrated that the Catboost model performed better in the prediction of surgical intervention in NEC between the above 2 models.

### 3.4 Establishment of the final model

**Figure 3:**
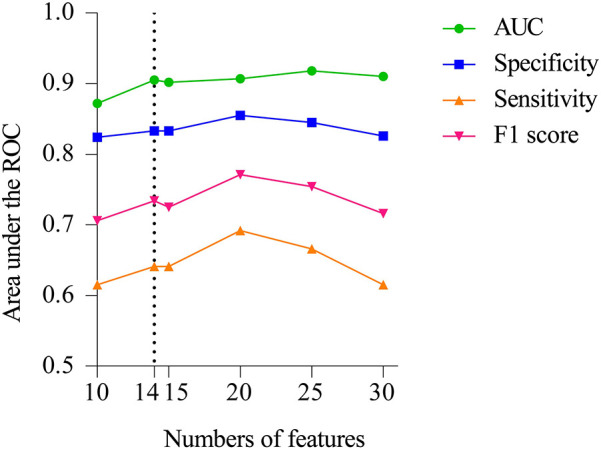
Performance of Catboost model to predict surgical NEC. AUC, sensitivity, specificity, and F1 score of the model with varied numbers of features are showed.

The final model was identified during the feature dimension reduction of the Catboost model. The 29 feature models were significantly better than the 10 feature models (AUC = 0.872) and 15 feature models (AUC = 0.902) in predicting NEC surgical intervention, but not significantly better than the 14 feature models (AUC = 0.905) in Figure 3. The 14 feature models have better net benefits and higher threshold probabilities compared with the 29 feature models. Therefore, our attention focused on the characteristics of the 14 Catboost model. These included base excess, WBC < 5 × 10^9^/L, vasoactive drugs before NEC, gestational age, serum albumin, sepsis, birth weight, PH < 7.25, hemoglobin, portal vein gas, feeding speed > 25 ml/(kg day), type-2 respiratory failure, use of drugs to close PDA before NEC, and frequent sleep apnea, which were used as the final model for further analysis. The AUC, specificity, sensitivity, precision, and F1 score of the Catboost model for predicting NEC were 0.905, 0.833, 0.641, 0.858 and 0.734, respectively.

### 3.5 The interpretation of the final model

**Figure 4:**
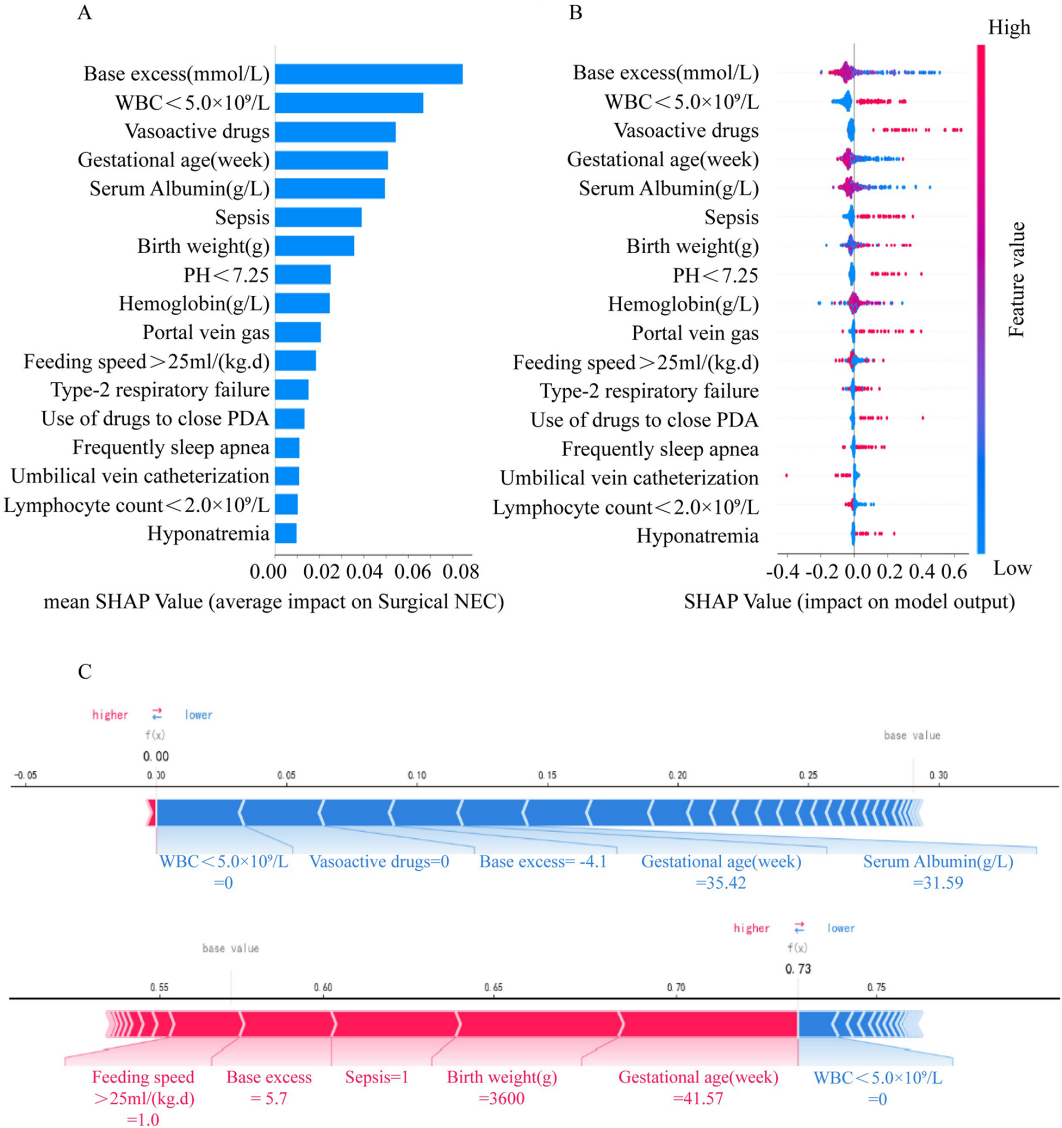
Interpretation of the effect of each feature on surgical NEC through SHAP values in the Catboost model. (**A**) SHAP summary bar plot. (**B**) SHAP summary dot plot. (**C**) SHAP force plot.

The output results of the final model were interpreted with the help of the SHAP method, and the average SHAP value was used to reflect the contribution of the features to the model. The influence of individual features on the model output and their importance ranking are shown in descending order in Figures 4A,B. Dot is made for SHAP value in the model for each infant, the colors of the dots demonstrate the actual values of the features for each one, as red means a higher feature value and blue means a lower feature value. The interpretation of the predictions using SHAP values are shown in Figure 4C. Red lines indicates features that drove a higher prediction probability of NEC surgery, and blue lines indicates features that drove a lower prediction probability. In addition, the length of the lever was proportional to the corresponding factors to predict the degree of contribution.

### 3.6 Model application

**Figure 5:**
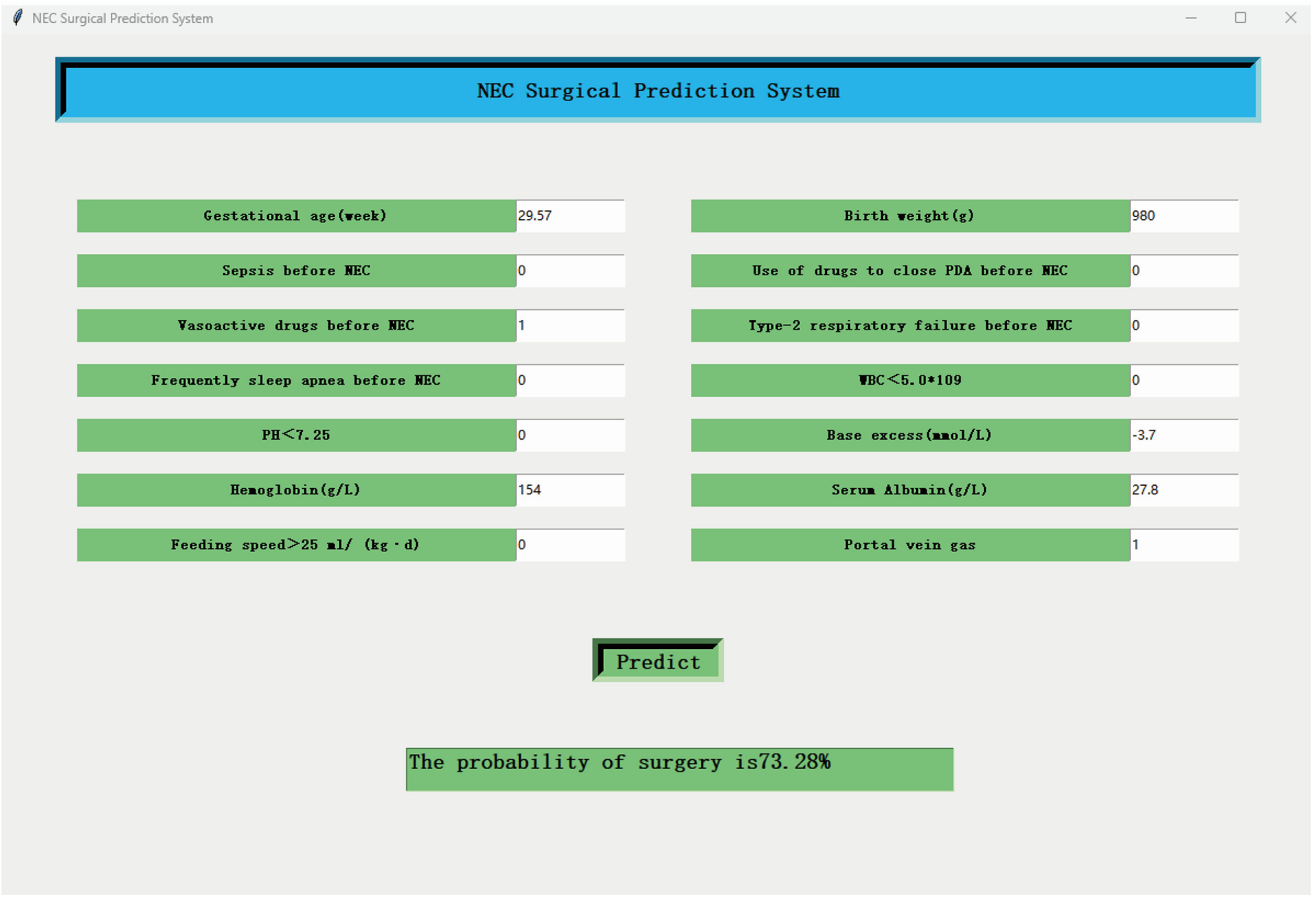
The screenshot of the GUI software. The final Catboost model with 14 features can be applied to surgical NEC prediction.

A Graphics User Interface (GUI) software was developed to facilitate clinicians to use the Catboost model. The GUI software screenshots are shown in Figure 5. The GUI software can be downloaded from https://nec-models.oss-cn-shanghai.aliyuncs.com/surgicalNECpredicted.exe and https://nec-models.oss-cn-shanghai.aliyuncs.com/model.pkl (please ensure that both files are placed in the same folder for the software to function properly). Clinicians or other users only need to input 14 feature values to directly obtain the prediction results to understand whether a child with NEC needs surgical intervention. This is beneficial to the choice of surgical timing and early intervention to improve the prognosis.

## 4 Discussion

There is a lack of specific treatment for NEC, and 20%–40% of the children with NEC require surgery. The mortality rate of children who underwent surgery is 50%, which is much higher than that of patients who underwent conservative treatment with internal medicine. In this study, 27.62% of children with NEC underwent surgery, and the postoperative mortality rate was 23.15%, which was significantly higher than that of the conservative group. Patients with NEC who underwent surgery, were more likely to have growth retardation and neurodevelopmental disorders (11). Early identification of NEC patients who may progress to surgery and timely intervention could reduce the percentage of surgical patients and minimize the risk of the disease to preterm infants. Machine algorithmic models minimized the covariate multicollinearity limitation and have greater advantages in clinical decision-making and early prediction compared with traditional logistic regression (12, 13). Several studies have explored machine learning models for predicting necrotizing enterocolitis (NEC); however, models specifically designed for predicting the need for surgery in NEC are relatively scarce. A study on deep learning using abdominal x-rays in NEC patients demonstrated that a ResNet18-based model could achieve an accuracy of 0.919 in diagnosing surgical NEC, highlighting its potential in optimizing surgical decision-making (14). This study undoubtedly reinforces the significance of abdominal x-ray in the assessment of NEC and surgical NEC. However, it is well recognized that the diagnosis of NEC and the decision regarding surgical intervention require a comprehensive clinical judgment from physicians, taking into account not only radiographic findings but also the overall clinical condition of the infant, physical examination, and laboratory results.

This study, identified 29 risk factors including perinatal period as risk factors for surgery in children with NEC through multifactorial analysis, but the covariance between variables was poor. The identification of the final model was carried out in the feature dimensionality reduction process of the Catboost model, and 14 risk factors were finally screened to construct the Catboost final model, including birth weight, gestational age, frequent sleep apnea, type II respiratory failure, use of drugs to close PDA, NRDS, sepsis, vasoactive drugs, WBC < 5 × 10^9^/L, hemoglobin, serum albumin, PH < 7.25, base excess, intestinal wall thickening, and portal vein gas. The Catboost model had better predictive efficacy for surgery in NEC patients than traditional multifactorial logistic regression analysis.

Previous studies showed that gestational age and birth weight are important risk factors for the occurrence of NEC and whether or not NEC patients are subjected to surgery (15, 16). It is well known that preterm infants with low birth weight have immature intestinal development and NEC is an ischemic and hypoxic injury of the intestinal mucosa due to multiple causes acting on the immature intestinal barrier (17). In addition, preterm birth and low birth weight may lead to reduced diversity of gut flora, and abnormal flora fixation (18). In this study, the NEC surgery group had a smaller gestational age and birth weight, which again confirmed that they were the key to understand whether or not a patient with NEC needed surgery.

Infection induces an excessive inflammatory response in the immature gut of preterm infants and plays an important role in the development of NEC. In this study, perinatal infection factors and related indicators (sepsis and WBC < 5 × 10^9^/L) were significantly higher in the NEC surgery group than in the conservative group. Bacterial circulatory translocation and associated signal transduction resulting from infection leads to increased intestinal mucosal damage and decreased perfusion, further leading to intestinal tissue necrosis (19, 20).

Hypoxia is likewise another important risk factor in the occurrence and severity of NEC. In neonates, especially preterm infants, with lower intestinal vascular resistance and poor ability to respond to and regulate systemic circulatory perturbations, redistribution of blood flow is more pronounced in a hypoxic-anoxic environment, further increasing the susceptibility of the intestinal mucosa to hypoxic-ischemic injury (21, 22). In this study, the surgery group had a higher rate of type-2 respiratory failure, frequent sleep apnea, PH < 7.25, use of vasoactive drugs, use of drugs to close PDA and lower hemoglobin and base excess levels.

Nutritional information and related indicators of patients were also included in this study, and nutritional factors were found to be involved in the progression of NEC, with serum albumin included in the Catboost optimal model. Albumin reflects the nutritional and immune status of the body to a certain extent, and low albumin levels often indicate poor nutritional and immune status (23, 24). Sharif et al. found that serum albumin ≤ 20 g/L on the day after the diagnosis of NEC was a good predictor of the need of surgery (25). Serum albumin after the onset of NEC was not measured in this study but the results showed that lower serum albumin levels in the early postnatal period could be associated with surgery in patients with NEC, suggesting that surgically treated patients have poorer nutritional and immune status in the early stages of life. In this study, the feeding speed >25 ml/(kg·day) was less common in the surgical group compared to the medical group before the onset of NEC. This finding likely reflects the underlying vulnerability of surgical group patients, such as a higher proportion of preterm infants, poorer baseline nutritional status, or higher risk factors for NEC. The lower feeding speed might have been a clinical precaution to minimize gastrointestinal stress in these high-risk infants. Although this observation seems to diverge from standard feeding practices, it underscores the importance of individualized feeding strategies in the management of high-risk infants.

The present model incorporated more comprehensive perinatal factors, including prenatal and postnatal clinical data, laboratory tests, and imaging data than other ML-based surgical prediction models for NEC. The risk factors included in the optimal model included baseline characteristics of preterm infants, infection, hypoxia, nutrition, and imaging, and the ROC value of the model was 0.905, which was significantly higher than that of the existing ML-based NEC surgical prediction models (8–10). In addition, a simpler GUI software was developed to improve the clinical tractability of the predictive model, enabling the early clinical prediction of performing or not the surgery in patients with NEC based on identified characteristic factors.

Despite the encouraging results, this study has some limitations. Firstly, this was a single-center study with a limited sample size and a long-time frame, which may have biased the results. In addition, although our model showed a good predictive efficacy, it has not been externally validated by recruiting patients from other clinical centers, and a prospective multicenter cohort study should be performed to optimize the model and validate it.

In conclusion, our work based on a decade-long retrospective study of NEC in Suzhou, China, revealed that 37.47% of children with NEC in our clinical center were subjected to surgery. In addition, 14 key characteristics associated with surgery of NEC patients were considered and a CatBoost model and simple GUI software were developed to predict whether a patient with NEC requires surgical treatment better than other existing prediction methods.

## Data Availability

The original contributions presented in the study are included in the article/Supplementary Material, further inquiries can be directed to the corresponding author.
